# Factors for Consideration in an Open-Flame Test for Assessing Fire Blocking Performance of Barrier Fabrics

**DOI:** 10.3390/polym8090342

**Published:** 2016-09-19

**Authors:** Shonali Nazaré, William M. Pitts, John Shields, Rick Davis

**Affiliations:** Fire Research Division, Engineering Laboratory, National Institute of Standards and Technology, Gaithersburg, MD 20899-8665, USA; william.pitts@nist.gov (W.M.P.); John.shields@nist.gov (J.S.); Rick.Davis@nist.gov (R.D.)

**Keywords:** residential upholstered furniture, barrier fabrics, flammability, thermal protective performance, thermal degradation, flaming ignition, flexible polyurethane foam

## Abstract

The main objective of the work reported here is to assess factors that could affect the outcome of a proposed open flame test for barrier fabrics (BF-open flame test). The BF-open flame test characterizes barrier effectiveness by monitoring the ignition of a flexible polyurethane foam (FPUF) layer placed in contact with the upper side of the barrier fabric, exposed to a burner flame from below. Particular attention is given to the factors that influence the ignitibility of the FPUF, including thermal resistance, permeability, and structural integrity of the barrier fabrics (BFs). A number of barrier fabrics, displaying a wide range of the properties, are tested with the BF-open flame test. Visual observations of the FPUF burning behavior and BF char patterns, in addition to heat flux measurements on the unexposed side of the barrier fabrics, are used to assess the protective performance of the BF specimen under the open flame test conditions. The temperature and heat transfer measurements on the unexposed side of the BF and subsequent ranking of BFs for their thermal protective performance suggest that the BF-open flame test does not differentiate barrier fabrics based on their heat transfer properties. A similar conclusion is reached with regard to BF permeability characterized at room temperature. However, the outcome of this BF-open flame test is found to be heavily influenced by the structural integrity of thermally degraded BF. The BF-open flame test, in its current form, only ignited FPUF when structural failure of the barrier was observed.

## 1. Introduction

The flammability of residential upholstered furniture (RUF) has long been recognized as a major contributor to residential fire losses in the United States and elsewhere [1] due to the rapid fire growth and high heat release rates frequently observed. As an example, fire statistics suggest that in the US fires involving RUF are responsible for 25% of all fire deaths in residences [2]. In 1975, California implemented Technical Bulletin (TB) 117-1975, which required that materials, such as polyurethane foam, used to fill furniture, be able to withstand a small open flame for at least 12 s [3]. Flame retardant (FR) chemicals were used widely in upholstered furniture to meet the FR standards of the California Bureau of Electronic and Appliance Repair, Home Furnishings, and Thermal Insulation’s (CBEARHFTI) Technical Bulletin (TB) 117 [4]. Due to the relatively large size of the California market and its influence on the overall furniture market in the United States, the use of flame retardants in RUF became common throughout the United States.

Concerns have been raised about the potential for these widely used FRs to have harmful human health and environmental effects [5,6]. Additionally, the effectiveness of FRs at the levels used in RUF for reducing RUF flammability was questioned [7]. The environmental concerns and the effectiveness of FRs used in RUF were publicized in a series of investigative articles in the Chicago Tribune [8]. In 2012, the governor of California requested the California Bureau of Electronics and Appliance Repair, Home Furnishing and Thermal Insulation (CBEARHFTI) to review the flaming ignition requirements of TB 117 [9]. As a result of this review, the flaming ignition test requirements were replaced by a test based on limiting cigarette (a smoldering source) ignition of upholstery-fabric covered flexible polyurethane foam (FPUF) [10].

Recent fire loss data has suggested that a very large percentage of the fire losses associated with RUF take place during flaming fires ignited by either smoldering or flaming sources [2,11]. Flaming fires initially ignited by a smoldering source occur following transition from a smoldering fire. Clearly, cost-effective alternative approaches for reducing fire growth rates and heat release rate levels in flaming fires involving RUF are desirable. The inclusion of a material (referred to as a barrier fabric) between the exterior upholstery fabric and the cushioning designed to slow ignition and/or limit the burning intensity has been suggested as a possible approach. Barrier fabrics are widely used to help meet mandatory flammability standards for mattresses [12] and California standards for reduced-flammability contract furniture [13].

Cal TB 117-2013 includes a provision that allows upholstery fabrics, which fail the smoldering ignition test, to be utilized when an appropriate barrier fabric, i.e., a material interposed between the outer upholstery fabric and the interior cushioning materials, is used to protect the interior cushioning materials from smoldering ignition. Recently, CBEARHFTI proposed an open-flame test [14] for barrier fabrics (BF-open flame test). The test method can provide insight into the response of barrier materials to an open-flame ignition source and the ability of the barrier fabric to prevent or slow down an external flame from reaching the FPUF and igniting it.

Recognizing the potential importance of barrier fabrics in RUF applications, researchers at the National Institute of Standards and Technology (NIST), have reviewed existing barrier fabrics and have developed potential methodologies for assessing the effectiveness of barrier fabrics in RUF fires ignited by flaming and smoldering sources [15,16,17]. However, several of the test methods that provide quantitative data use expensive equipment and require skilled personnel, both of which are not always easily available to the test facilities in the industry. Protective performance testing of barrier fabrics must be as simple and inexpensive as possible, while still providing science-based information. The open flame test drafted by CBEARHFTI for barrier fabrics, proposes a possible standard testing method with a goal of producing upholstered furniture which is safer from the hazards associated with small open-flame ignition [14].

The BF-open flame test used in this study, which mirrors the CBEARHFTI proposed standard testing methods, has some similarities to ASTM D7140, Standard Test Method to Measure Heat Transfer through Textile Thermal Barrier Materials [18]. ASTM D7140 is designed to measure heat transfer through textile materials and to differentiate barrier materials based on their heat transfer properties. In this test, the barrier material is exposed to a well-defined and controlled convective heat source (propane flame from a Meker burner) from underneath for 60 s. The temperature on the unexposed side of the test specimen is monitored. This test method essentially produces a time-temperature curve; however, it does not define a heat transfer threshold for textile materials that can be used as a criterion for fire barrier materials.

**Figure 1:**
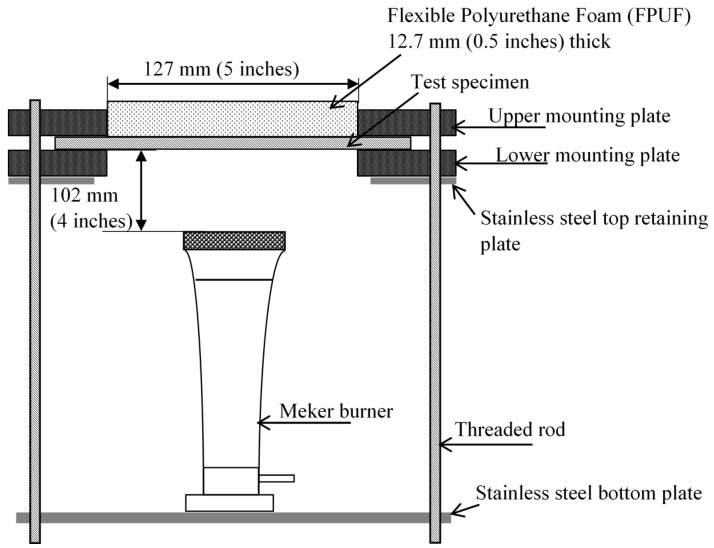
BF-open flame test apparatus.

The BF-open flame test method consists of a similar application of a pre-mixed butane flame to the underside of a horizontally mounted specimen of the barrier fabric (see Figure 1). However, unlike in ASTM D7410, a layer (12.7 mm thick) of non-FR FPUF, having specified properties, is placed on the unexposed side of the barrier fabrics away from the flame. The test specimen is sandwiched over an opening formed by two rigid fire-rated insulating boards supported by a metal rack. The barrier fabric (BF) specimen fails the test if the foam on top of the BF specimen ignites. The differences and similarities between ASTM D7140 and the BF-open flame test are given in Table 1. The amount of material required and the exposure area of the test specimen are larger in the case of the BF-open flame test. Both test methods use the same types of Meker burner, and the flame application times are identical. The distance between the top of the burner and the test specimen is almost double for the BF-open flame test than in the ASTM D7140 test method. The gas flow rate is specified in the BF-open flame test, whereas in the ASTM D7140 test method the gas flow rate is adjusted to obtain a heat flux of 46 kW/m^2^ from the flame exposure. ASTM D7140 requires the use of a propane flame, while the BF-open flame test uses a butane flame.

ASTM D7140 measures the heat penetration through a BF when exposed to an open flame. The BF-open flame test, on the other hand, assesses barrier effectiveness by monitoring the ignition of a FPUF layer placed in contact with the unexposed side of the barrier fabric. Thus, while ASTM D7140 is a quantitative test, the BF-open flame ignition test is a pass/fail test. Moreover, at first glance the BF properties responsible for limiting the ignition of FPUF in BF-open flame tests are uncertain. In this study, we examine the factors that influence the ignitability of FPUF in the BF-open flame test method. The BF-open flame test method was evaluated by comparing the results of several commercially available barrier fabrics from the BF-open flame test. Additionally, measurements of heat transfer through the BFs were made using a technique similar to that described in ASTM D7140. Observations of the FPUF burning behavior and char patterns in the BF-open flame test are included in the assessment of the performance of the BF specimen under the BF-open-flame test conditions.

## 2. Materials and Methods

Certain commercial equipment, instruments or materials are identified in this paper in order to specify the experimental procedure adequately. Such identification is not intended to imply recommendation or endorsement by the National Institute of Standards and Technology, nor is it intended to imply that the materials or equipment identified are necessarily the best available for this purpose.

### 2.1. Materials

The sample descriptions and physical properties of 17 commercially available BFs tested during the current study are given in Table 2. For comparison purposes, the sample identification numbers have been kept identical to those used in our previous studies [16,17]. New BFs included in this study were assigned higher numbers. The exclusion of BFs studied earlier from the current study was either due to unavailability of sufficient barrier fabric (BF-6, BF-7, and BF-14) or to the similarity of fabric structures (BF-11, BF-12 both knitted and similar to BF-13 and BF-17, BF-18 both woven glass fabrics and similar to BF-19). The list includes a variety of textile structures including high-loft, nonwoven battings, knitted, woven structures, and back-coated fabrics. The BFs varied in average thicknesses from 0.1 mm to 7.8 mm. The experimental matrix covers the most extensively used fibers and fiber blends in the BF industry. These include flame retardant (FR) rayon, low-melt polyester, FR polyester, glass fiber, aramid fibers, and blends thereof. BFs made from the latest core-yarn technology and high-performing polyaramid/melamine fiber blends are also included. The exact fiber blend compositions are proprietary and thus were not available.

Depending on the mode of fire blocking technology employed, the BFs in Table 2 are identified as passive or active. By providing a physical barrier between the heat source and the cushioning material, passive barriers can limit pyrolysis and heat release rates. Their effectiveness derives from serving as a physical and/or thermal barrier between some or all of the fuel and the potential ignition source. Active BFs have a chemical effect on the fire. Active flame retardants may act in the condensed (or solid) phase or in the gas phase or as a combination of both. In the condensed phase, the FR action typically results in enhanced char formation whereas in the gas phase the FR undergoes thermal breakdown to form compounds that can lead to flame quenching.

BFs can also be distinguished as thermally thick or thermally thin materials. These terms come from heat transfer analysis and refer to whether or not temperatures inside a material vary spatially, when the surface is exposed to a heat source or sink. In thermally thin materials, heat transfer occurs easily and quickly through the material. In these systems, the temperature on the outside of the object is roughly the same as the temperature throughout the material; i.e., there are minimal temperature gradients inside the material [19]. In contrast, temperatures inside thermally thick materials can vary widely, when exposed to a heat source because of their relatively high level of heat transfer resistance. In fabrics, thermal thickness is a function of heat transfer resistance relative to the physical thickness of the fabric [20].

Considering the air permeability properties in Table 2, BFs can be characterised as permeable and impermeable barriers. For the purpose of this study, impermeable barriers are defined as barriers with air permeabilities (measured with a target pressure drop of 125 Pa [17]) less than 1 m/s. These impermeable barriers limit the transport of volatile gases through the barrier.

Non-flame retarded FPUF pieces of known dimensions meeting Cal TB 117-2013 [10] were procured from Innocor Foam Technologies, Coldwater, MS, USA.

### 2.2. BF-Open Flame Test

A schematic of the BF-open flame test apparatus used in this study is shown in Figure 1. A BF specimen (approximately 250 mm × 250 mm) was sandwiched between two rigid fire-rated insulating boards, each equipped with a square (127 mm × 127 mm) opening, supported by four supporting threaded rods. A piece (127 mm × 127 mm × 12.7 mm) of FPUF was placed in the opening directly above and in contact with the BF test specimen. A Meker burner (Humbolt Manufacturing Co. Part number H-5600, Elgin, IL, USA) with a top diameter of 32 mm and orifice size of 1.2 mm was used. A butane flame was used with a fuel flow rate of 500 ± 10 mL/min at ambient conditions, while keeping the venturi throat in a fully open position [23]. The flame was applied to the test specimen from underneath. The Meker burner was manually placed at the center of the bottom plate of the test rig such that the top of the burner was positioned 102 mm below the center of the bottom surface of the test specimen (Figure 1). The flame was applied for a period of 60 s. A barrier fabric failed the test if flaming ignition of the FPUF was observed in any of three repeated runs for a given barrier.

### 2.3. Temperature and Heat Transfer Measurements

The experimental setup for measurement of heat transfer through the barrier fabrics was the same as shown in Figure 1 except that the piece of FPUF was replaced by a slug calorimeter (Model: ST-8-21915 S/N 169222, Medtherm Corporation, Huntsville, AL, USA) embedded in an insulating board and placed face down on the BF specimen. This is similar, but not exactly the same as the set-up defined by ASTM D 7140. The slug calorimeter consisted of a blackened copper disc 40 mm in diameter with a thickness of 1.6 mm. Three 32-gauge Type K chromel/alumel thermocouples were mounted along the perimeter of the disk at 120° locations. The calibrated slug calorimeter was connected to a data acquisition system which recorded the rise in temperature of the sensors as a function of time. The rates of temperature rise were used in conjunction with the calorimeter constants provided by the manufacturer to compute the heat flux received. At the start of the test, the heat sensors were approximately at room temperature. A particularly useful feature of this test procedure is a continuous calorimetric trace useful for analyzing the fabric heat transfer characteristics.

The heat flux from the flame exposure was calibrated by placing the slug calorimeter facing down so that it was exposed directly to the flame. The distance between the bottom of the calorimeter and the burner top was kept at an approximate distance of 102 mm. The gas flow rate of butane through the flow meter was maintained at 500 ± 10 mL/min. The heat flux from the butane flame was measured as 50 ± 3 kW/m^2^. This is comparable to the heat flux (46 kW/m^2^) obtained using propane gas as specified in ASTM D 7140 test method.

## 3. Results and Discussion

### 3.1. General Experimental Observations during BF-Open Flame Test

As mentioned in the experimental section, during the BF-open flame test the open flame impinges directly on the BF at the center. Depending on the type, the barrier fabric ignites or undergoes charring. Heat transferred through the BF heats the foam and, if high enough, can cause the foam to undergo thermal decomposition.

**Figure 2:**
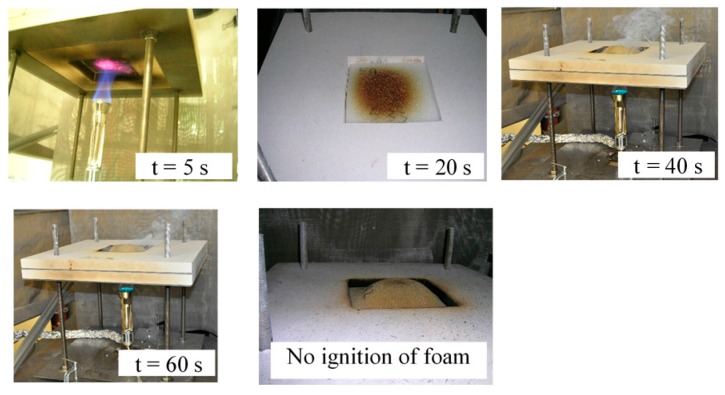
Digital images showing a thermally thick barrier fabric (BF-3) exposure to the open-flame ignition source, dome formation, and no ignition of foam.

As the temperature increases, the foam undergoes both physical and chemical changes. Physically, the foam is observed to deform and move away from the heat source, resulting in an increased surface area. The FPUF forms a dome-like structure such as the one visible in Figure 2. The center of the FPUF dome is generally positioned over the tip of the applied flame. Such physical changes in the FPUF may change the heat transfer between the BF and the FPUF and may have a significant effect on subsequent decomposition processes of the foam.

When heated, the FPUF can initially undergo two main reaction processes. One pathway is a pyrolysis involving the reverse of the polymerization process, leading to the formation of toluene diisocyanate (TDI)-derived gaseous products and polyol-derived liquids [24]. The second is an oxidation process that produces a char. The composition of degradation products depends very much on the conditions of pyrolysis [25]. During the first stage of pyrolysis, the heated foam releases gaseous products (usually seen as a yellow smoke) from the isocyanate component of the foam formulation [26] and is probably the most volatile fraction of the released species [24]. However, this gaseous fuel does not necessarily ignite in the BF-open flame test; most probably due to lack of sufficient heating and/or an ignition source. Careful inspection of residual materials after the test, especially where ignition of FPUF did not occur, revealed that a thin layer of liquid, essentially regenerated polyol, was formed on the surface of FPUF that was in contact with the BF. Based on our previous observations of FPUF pyrolysis and/or burning behavior, formation of a thin layer of liquid, believed to be regenerated polyol, is consistent with current observations. Further heating led to pyrolysis of this materials.

**Figure 3:**
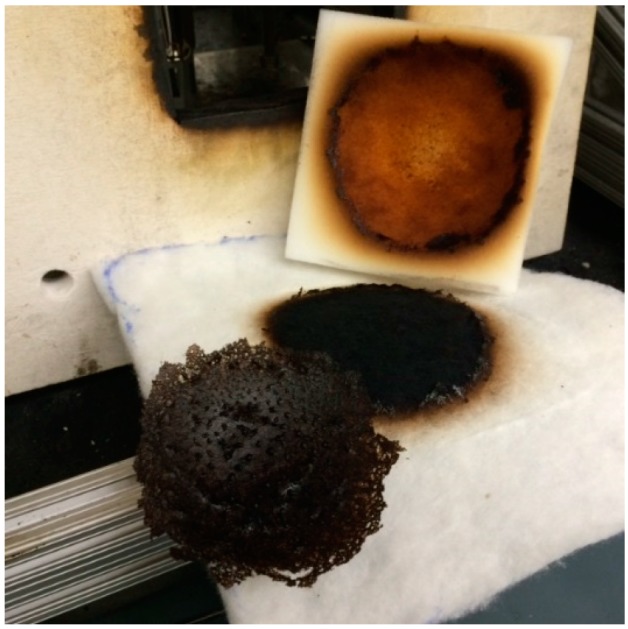
Digital image of char removed from the FPUF tested with BF-2.

In the presence of thermally thick barriers, where the rate of FPUF heating is much slower compared to that for thermally thin barriers, the FPUF decomposition is more likely to be dominated by the second pathway, whereby oxidation reactions produce significant char. Highly porous, thermally insulating highloft barriers provide appropriate conditions, i.e., low heat losses and sufficient oxygen supply to allow the FPUF undergo significant low-temperature smoldering combustion. An example of the char formed during this type of non-flaming mode of combustion is shown in Figure 3.

As heating from the bottom continued, holes were seen to be formed in the dome structure due to foam pyrolysis. This caused the dome structure to collapse. This phenomenon was generally observed in cases of thermally thin BFs (BF-8, BF-9, BF-10, BF-13, BF-15, BF-19, BF-20, BF-21, BF-22 and BF-23) suggesting that for these tests substantial amounts of heat were transferred to the foam in a very short period. Gaseous products were visually observed being released immediately following the hole formation. The volatilized gases came into contact with fresh air and although the pyrolyzed foam and BF surface may have been at a high temperature, flaming ignition was not observed. The thermal degradation of FPUF and subsequent ignition requires a mixture of combustible gases and air that are within the flammability limits and also a sufficient heat source for the mixture to initiate exothermic reactions and ignite [27,28]. In this open configuration, heat losses and the escape of gaseous products to the surroundings occur at high rates which could be the explanation for the failure of pyrolysis gases to auto-ignite.

**Figure 4:**
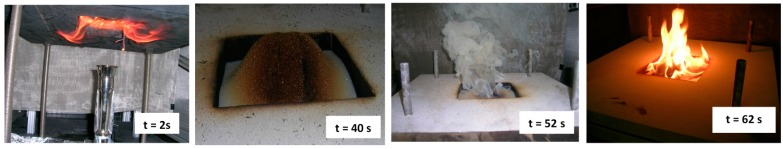
Digital images showing stages of a thermally thin, highly permeable barrier (BF-1) exposure to the open-flame ignition source, flaming of BF-1, FPUF degradation and dome formation, dome collapse and release of gaseous products, and flaming ignition of FPUF following the opening of a direct pathway through the BF.

Flaming ignition of the foam was only observed when the BF char cracked open and flames from the burner came into direct contact with material on top of the barrier. When flaming ignition occurred the barrier failed the test. Following ignition the complete piece of foam was engulfed in flames. Digital images of BF exposure to the open-flame ignition source, ignition and burning of BF, FPUF degradation and subsequent ignition of FPUF following crack formation in the BF are shown in Figure 4.

**Figure 5:**
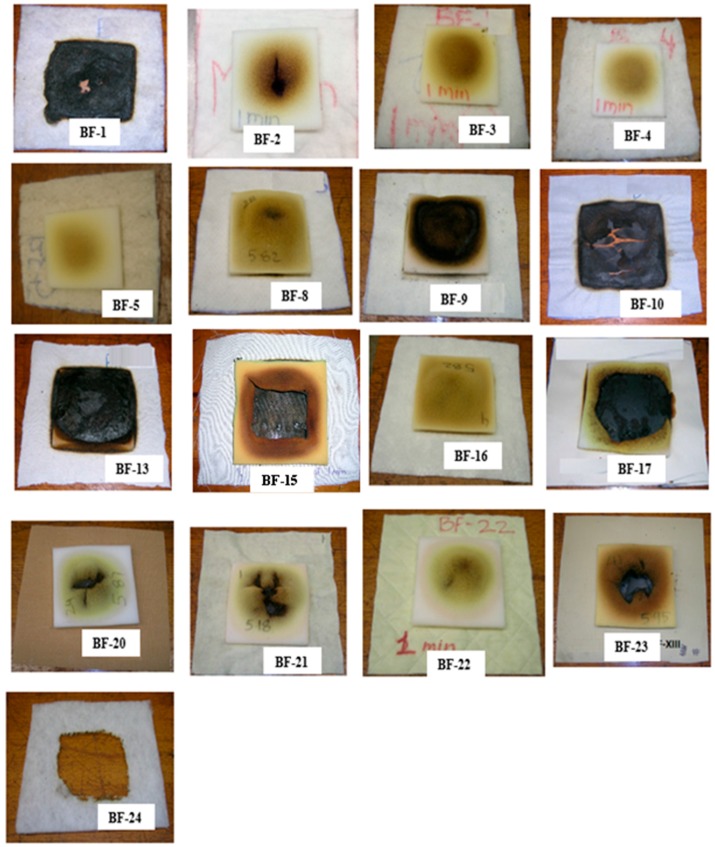
Digital images of unexposed sides of barrier fabrics and FPUF specimens (12.7 mm thick) after 60 s of exposure to open flame ignition source.

A variety of FPUF behaviors are observed depending on the barrier properties, e.g., thermal thickness, gas permeability, flammability, and structural integrity. Images of barrier fabrics and FPUF specimens (12.7 mm thick) after a 60 s exposure to the open flame ignition source are shown in Figure 5. Only three BFs; BF-1, BF-10, and BF-24 failed this test. For the BFs that failed the BF-open flame test, holes or crack formation in the BFs can be clearly seen. The images for BF-13, BF-15, and BF-17 in Figure 5 clearly show that the FPUF was significantly pyrolysed; however, no flaming ignition of FPUF was observed during the test duration of 60 s. The test duration of 60 s was not sufficient to form openings (holes/cracks) in the BFs and the use of 12.7 mm (1/2″) thick foam provided insufficient fuel to pyrolyze beyond the 60 s test duration. Thus, the BF-open flame test method, does not create flammable conditions despite high rates of FPUF pyrolysis. The test results also seem to depend on whether or not FPUF is fully decomposed prior to barrier breakthrough. In order to provide additional insight into the test behavior, these BFs were retested using 25.4 mm (1″) thick foam pieces. The modified BF-open flame test was terminated after 5 min of flame application if no ignition of FPUF was observed. Table 3 shows the results of the BF-open flame test with 12.7 mm FPUF and the modified BF-open flame test with 25.4 mm FPUF. An additional four BFs (BF-2, BF-13, BF-15, and BF-20) failed the modified open flame test. Again, the FPUF ignition occurred due to direct flame impingement on the FPUF through openings formed in the BF.

The following section describes the effects of BF properties on thermal decomposition of FPUF in the BF-open flame test.

#### 3.1.1. Thermally Insulating Barriers

Highloft nonwoven BFs are characterized by high volumes of air that exceed the volume of fiber. BF-1, BF-2, BF-3, BF-4, BF-5, and BF-24 are highloft barriers. BF-1, BF-2, BF-3, BF-4, and BF-5 are made up of char-forming fiber blends. These BFs vary in area densities, thicknesses, and gas permeabilities. All of these BFs passed the BF-open flame test except BF-1. All of the tested highloft barriers ignited and burned briefly when exposed to the open flame ignition source. BF-1 burned and formed a very fragile char with holes in it. The flames from the ignition source reached the flammable material above the barrier, which caused FPUF ignition. BF-1 thus failed the BF-open flame test. The lower area density and thickness in combination with higher permeability of BF-1 compared to the other highloft barriers likely explain its poor performance.

In the case of BF-2, BF-3, BF-4, and BF-5, the thermally insulating carbonaceous char was formed following the ignition and/or thermal degradation of component fibers. The carbonaceous thick char enhances flame and heat resistance. Moreover, the char was found to be structurally intact with no holes or cracks, thus preventing the burner flames from reaching the FPUF. The undamaged dome structure of the FPUF surface, particularly for BF-3, BF-4, and BF-5 (see Figure 5), indicated that heat transfer into the FPUF was limited by the thermally thick barrier. In an attempt to assess the robustness of this carbonaceous insulative char, we ran additional tests; whereby FPUF thickness was increased from 12.7 mm to 25.4 mm and flame was applied until ignition of FPUF was observed. The times to ignition of FPUF are given in Table 3. It was noted that the FPUF ignited only when a hole or a crack was formed in the barrier.

BF-24 is comprised of 100% polyester fibers and is generally referred to as polyester batting. Traditionally, this material has been used in upholstered furniture to give superior appearance and performance characteristics to the finished product. In residential mattresses, a thin layer of such polyester batting is often used to enhance smoldering ignition resistance. A similar approach has been suggested for upholstered furniture whereby a polyester batting could act as a barrier to a smoldering ignition source [29,30,31]. However, this material fails the BF-open flame test as the thermoplastic polyester melts as soon as it is exposed to this open-flame ignition source. As the polyester batting melts and shrinks to form a large hole (Figure 5), the FPUF placed directly above is exposed to the flaming ignition source. The FPUF ignites and burns completely, thus failing the test.

#### 3.1.2. Impermeable or Barriers with Low Gas Permeability

Needle-punched barriers are nonwoven materials characterized by high area density and low thickness, resulting in higher bulk densities (see Table 2). These barriers also have very low air permeabilities. BF-8, BF-9, and BF-16 in Table 2 are characterized as needle-punched flat barriers. These barriers protect the FPUF sufficiently well to pass the BF-open flame test. In fact, the char of the thermally degraded barriers was structurally intact even after 5 min of exposure to the open flame ignition source.

BF-17, BF-20, BF-22, and BF-23 are additional BFs with very low or zero air permeabilities. Examples of images of residual FPUF char taken after completion of these test are shown in Figure 5. Even though the FPUF shows signs of thermal degradation, there is no trace of ignition and these BFs passed the BF-open flame test. These BFs remain structurally intact, i.e., no hole or crack formation, and the flame does not come into contact with the FPUF or the volatiles.

It is interesting to note that the FPUF does not form a uniform dome-like structure in the presence of thermally thin, gas impermeable, high thermal conductivity barriers, for example, BF-17. The FPUF forms a transient dome-like structure, however, further heating and rapid heat transfer via conduction and radiation, results in faster decomposition of FPUF and a subsequent collapse of the dome-like structure. The images of residual char in Figure 5 suggest that the FPUF above BF-17 pyrolyzed to a greater extent when compared to those on top of BF-20, BF-22, and BF-23. This can be associated with the thickness of the barrier. BF-17 is almost half the thickness of BF-20 and therefore, a higher heat transfer rate is to be expected. The heat flux measurements on the unexposed side of these BFs, discussed in the sections below, showed that the total amount of heat transferred through BF-17 is indeed higher than that through BF-20, BF-22, and BF-23. In the case of BF-23, which is a BF with flame retardant back-coating, some of the heat from the flaming ignition source is dissipated in the endothermic reaction of the FR back coating.

#### 3.1.3. Thermally Thin, Permeable Barriers

**Figure 6:**
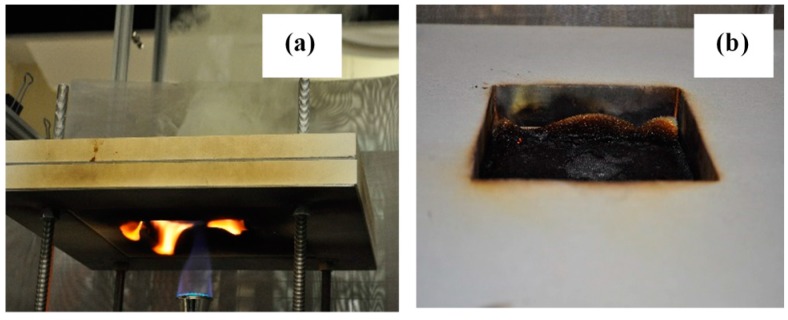
Digital images showing (**a**) flaming of liquefied FPUF below BF-15 and (**b**) unburned, thermally degraded FPUF.

BF-13, BF-15 and BF-21 are thermally thin, highly permeable barrier fabrics. BF-13 is a knitted fabric, and BF-15 is a woven fabric, both made from core-spun yarn. BF-21 is a nonwoven fabric comprised of fire resistant para-aramid fibers. These BFs pass the BF-open flame test, however, BF-13 and B-15 failed the longer exposure open flame test. In the case of these thermally thin, permeable BFs, the FPUF dome structure formed and collapsed as holes formed in the FPUF. At the same time, rapid heat transfer through the thermally thin, permeable BFs led to rapid decomposition of FPUF. Liquefied FPUF dripped away from the FPUF and formed a pool of low viscous flammable liquid on top of the BF. In some of the tests, liquid FPUF was actually observed to seep downward through the barrier material and burn on the burner side of the barrier. Since the FPUF did not ignite or burn above the barrier during the test period, the BF-13 and BF-15 (see Figure 6) met the BF-open flame test criterion even though flames were clearly generated from material associated with the FPUF. For BF-13 and BF-15, dripping of liquefied FPUF was beneficial in terms of ignition resistance above the barrier fabric. BF-21, a thermally thin, non-woven material with gas permeability comparable to BF-15 (2.1 ± 0.1 m/s), also passed the BF-open flame test. In the case of BF-21, the FPUF undergoes little degradation. The FPUF formed smaller amounts of the liquid decomposition product, as seen for BF-13 and BF-15. This is remarkable in that the initial physical properties of these BFs (BF-13, BF-15 and BF-21) are comparable. A possible explanation is that heating of the FPUF can be different for BF-13 and BF-15 as the BF properties e.g., gas permeability, are likely to change due to thermal degradation of the constituent fibers. The fire resistant para-aramid fibers in BF-21 char in place and therefore the gas permeability may remain unaltered.

It can be noted from Table 3 that ignition times for BF-15 in the modified BF-open flame test were in excess of 60 s. A knitted fabric with a glass-fiber blend (BF-13) and nonwoven para-aramid barrier (BF-21) both failed to protect the FPUF from heat and flames in the modified BF-open flame test. When exposed to an open flame for longer duration, holes are formed in the thin BFs, through which the flames from the ignition source penetrated and ignited the FPUF.

As discussed, not all BFs succeed in protecting the FPUF from ignition. The level of protection depends on complex relationships between the heat transfer properties, gas permeabilities of the BF, and structural integrity of BF char. The properties of barrier fabrics are governed by the fiber type and construction. In order to understand the thermal degradation of the FPUF and heat fluxes, to which the FPUF is exposed, we measured temperatures and heat fluxes on the unexposed sides of barrier fabrics using the instrumentation described earlier. The following section describes the heat transfer properties of the BFs tested in the BF-open flame test configuration.

### 3.2. Heat Flux Measurements on the Unexposed Side of BFs

In order to compare the results of the BF-open flame test to the heat transfer properties of the BFs, the heat flux on the unexposed side of the BF was measured using the technique described in Section 2.3. The heat flux recorded during the 60 s exposure time and the total amount of heat transferred (THT) through the BF at the end of the 60 s test duration are given in Table 4. Uncertainties in measurement of various heat flux related parameters are reported as Type A uncertainties [21,22] in Table 4. A Type A evaluation of standard uncertainty is determined as the standard deviation of a series of independent observations. Descriptions of the BF chars are also included in Table 4. It is evident from the char description that the BFs that fail in the BF-open flame test, fail as the result of structural failure of the barriers.

**Figure 7:**
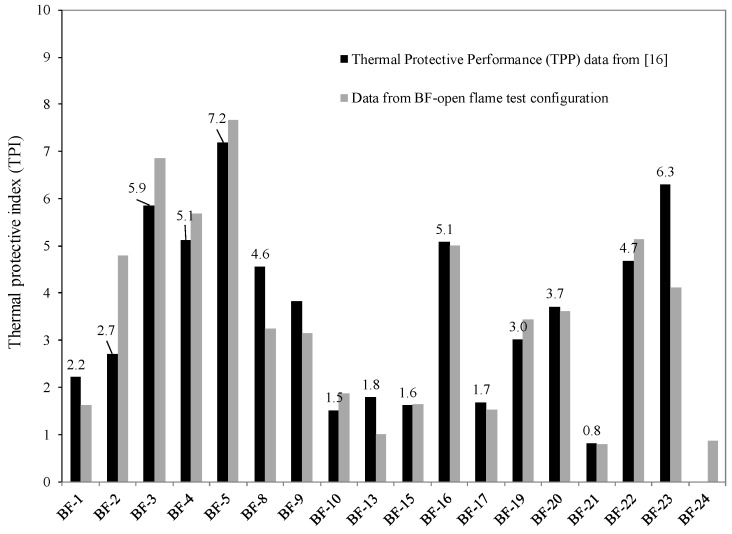
Comparison of BF rankings using TPI from two different test configurations.

Derived quantities such as the heat transfer factor (HTF) and thermal protective indices (TPI) given in Table 4 and plotted in Figure 7, respectively, have been used to characterize the heat transfer properties of the barrier fabrics. The HTF is a mass normalized THT value having units of J/g. HTF is the ratio of the total heat transferred in MJ/m^2^ (THT) value to the fabric area density in g/m^2^. The inverse of the HTF was previously defined as the thermal protective index (TPI) for a BF and was used to rank BFs for their thermal protective performances [16]. The ranking of BFs using thermal protective indices derived from heat transfer data obtained from the thermal protective performance (TPP) test device [16] and the BF-open flame test setup are plotted in Figure 7. The ranking order is almost the same for both sets of data. The higher the TPI value, the better the thermal protective performance of a BF.

The experimental data suggest that different barriers have different heat transfer properties such that the total amount of heat transferred through the barriers is “different” and the maximum heat flux measured on the unexposed side of the barrier also varies with the type of barrier.

**Figure 8:**
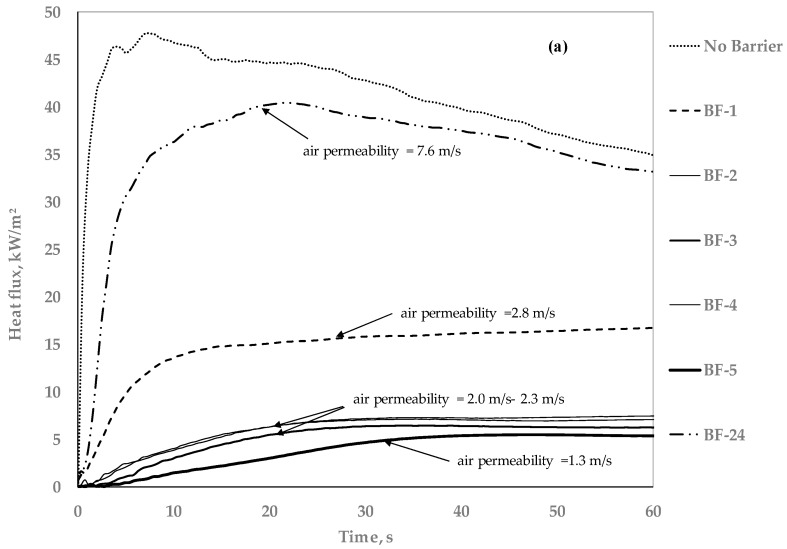

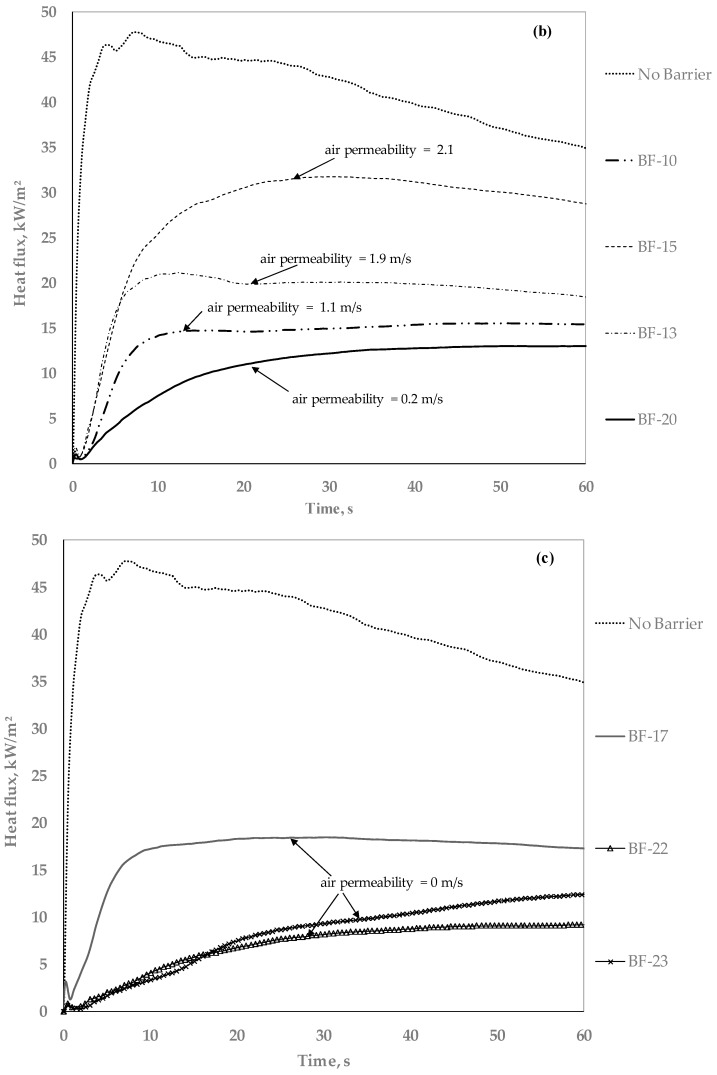

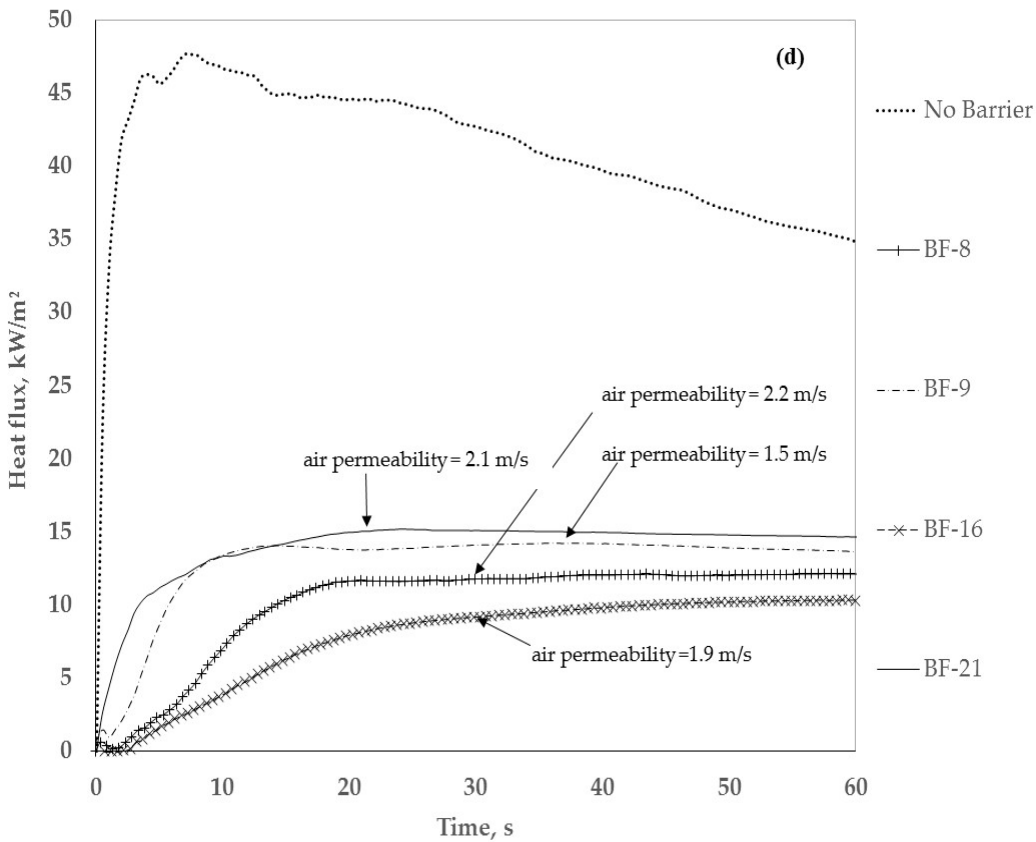
Thermal response of (**a**) thermally thick, highloft; (**b**) thermally thin, permeable; (**c**) thermally thin, impermeable; and (**d**) flat non-woven BFs.

The degree of thermal protection provided by the barrier fabrics can also be assessed by monitoring the heat flux versus time data collected during the tests run with the slug calorimeter (see Figure 8). Thermal responses of (a) thermally thick, highloft; (b) thermally thin, permeable; (c) thermally thin, impermeable; and (d) flat non-woven BFs are shown in Figure 8.

The heat flux versus time curve for direct slug calorimeter exposure (without barrier fabric) is included for reference. It can be seen that high heat flux values of 45 ± 5 kW/m^2^ are reached almost instantaneously in the absence of a barrier. Immediately after the peak is reached, the heat flux curve starts to decline. The decrease in the heat flux measurement could be due to decreasing the difference between the heat source and the surface temperature of the slug and/or due to heat losses from the slug to its holder [32]. Correcting the slug calorimeter results for such effects in order to achieve more accuracy is beyond the scope of this work. It is important to note here that the heat flux data is used to compare the heat transfer properties of different barriers. It is also worth mentioning that the heat flux versus time curves reach steady states after a peak is reached in the presence of a barrier, suggesting no additional changes in barrier effectiveness with time. Generally, it can be seen that only a third of the applied heat flux is transferred through a char forming barrier.

There are no great differences in the BF behavior during heat flux measurements as compared to the BF-open flame test with FPUF. For example, BF-24 melted and shrank away from the flame, BF-10 formed cracks, and BF-1 had holes in it as described in Table 4. However, none of these BFs show an instantaneous increase in heat flux on the formation of cracks or holes except BF-24, for which the heat flux increases significantly as soon as the polyester batting melts and forms a hole to expose the slug calorimeter to the flame. As discussed earlier, the FPUF ignites, and BF-24 fails the test. Comparing heat flux versus time curves for the BFs that failed the test, it can be noted from Figure 8 that BFs can fail the test without reaching high heat flux values, i.e., the barriers fail due to structural failure. The FPUF ignites only when the flames from ignition source come into contact with volatiles. The transient response of the slug calorimeter to an instantaneously imposed high heat flux is determined by a thermal energy balance of the slug [33] and solicits further studies to investigate instantaneous heat transfer due to structural failure of the BFs.

## 4. Conclusions

The BF-open flame test is an easy to operate, cost-effective test method designed to assess the protective performance of fire blocking barrier fabrics when exposed to a flaming ignition source. Several commercially available barrier fabrics were tested using the proposed test method. In the BF-open flame test, ignition of the FPUF is only observed when the BF breaks open and flames from the ignition source come into direct contact with decomposing FPUF or its volatiles. Once flaming ignition occurs, the complete piece of foam is engulfed in flames, and the barrier fabric fails the test. However, if the BFs remain intact, the foam does not ignite, resulting in the barrier fabric passing the test. Thus, the BF-open flame test that was considered in this study only differentiates barriers based on their structural integrity. However, relative barrier fabric effectiveness is known to vary with such properties as flammability, thermal resistance, and permeability, in addition to structural integrity [16]. Since the BF-open flame test method seems to be primarily sensitive to barrier structural integrity, it does not differentiate in terms of other important properties of BFs described earlier in [16].

Heat flux measurements suggested that roughly only one third of heat from the open flame ignition source is transferred through char forming barriers. Moreover, it was observed that the BFs can fail the test without reaching high heat flux values, i.e., the barriers fail primarily due to structural failure. In many cases the FPUF pyrolysed significantly however, the test set up does not create flammable conditions despite high rates of FPUF pyrolysis as long as the barrier remains intact. The 12.5 mm thick foam used in the BF-open flame test method provides a relatively small quantity of fuel that is, in some cases, either fully pyrolysed or charred before the end of the test duration of 60 s. A larger number of BFs failed in protecting the FPUF beyond the 60 s test duration when the FPUF thickness was increased from 12.5 mm to 25 mm. However, ignition times were significantly longer and FPUF ignitions still occurred due to hole/crack formation in the BF, suggesting that there exists a time factor in the ability of the BF to protect the foam based on the time to structural failure of the barrier.

The inverted test configuration with the FPUF placed on top of the BF as opposed to real furnishing configuration, where the FPUF would be located under the fire blocking barrier fabric, presents an implausible challenge. There may be burning scenarios where cushion could be insulted by flames from below, but the reverse seems much more probable. In a real furnishing configuration, the FPUF would pyrolyze and collapse when exposed to heat and flames. In the BF-open flame test configuration, the FPUF often swells away from the ignition source, forming a dome-shaped structure over the BF. The dome formation is a result of a specific test configuration and this would not happen in the case of a real furniture fire. The shape and volume of the dome structure formed due to thermal decomposition of the foam sample varied strongly with the type of BF. The variation in size and shape likely caused a significant variation of flame heat transfer to the FPUF, which would strongly affect the likelihood of FPUF ignition in real-world configurations.

Based on this limited experimental series, while open flame test method offers several advantages with regard to its simplicity and ease of use, the BF-open flame test used in this study appears to assess barrier fabrics with regard to their resistance to breaking open and exposing the protected material to direct flame impingement. Since BF effectiveness is known to be determined by a number of other parameters [16], it may be necessary to revise the BF-open flame test methodology to more adequately replicate BF performance in actual applications.

## Figures and Tables

**Table 1:** Comparison of ASTM 7140 and BF-open flame tests.

Description	ASTM 7140	BF-open flame test
Test specimen	133 mm × 133 mm	250 mm × 250 mm
Exposed specimen area	76 mm × 76 mm	127 mm × 127 mm
Burner	Meker burner 38 mm diameter, 1.2 mm orifice size	Meker burner 38 mm diameter, 1.2 mm orifice size
Gas	Natural gas/propane	Butane
Gas flow rate	Not specified	500 ± 10 mL/min
Distance between specimen and burner	50 ± 1.6 mm	100 mm
Flame application time	60 s	60 s
Heat flux of flame exposure	46 kW/m^2^	Not specified

**Table 2:** Description and physical properties of barrier fabrics.

ID	Fiber blend	Structure	FR system	Thickness (mm)	Area density ^§^ (g/m^2^)	Bulk density (g/cm^3^)	Air permeability (m/s)
BF-1	Flame retarded (FR) rayon/polyester	Highloft	Passive	4.1 ± 0.1	155	0.038	2.8 ± 0.2
BF-2		Highloft	Passive	6.7 ± 0.2	230	0.034	2.0 ± 0.1
BF-3		Needle punched	Passive	7.8 ± 0.6	240	0.031	2.3 ± 0.1
BF-4	Boric acid treated cotton/FR rayon/polyester	Needle punched/Stratified	Passive	5.7 ± 0.1	230	0.040	2.2 ± 0.2
BF-5	Boric acid treated cotton	Needle punched	Passive	6.9 ± 0.8	230	0.033	1.3 ± 0.1
BF-8	FR rayon/polyester	Needle punched nonwoven	Passive	4.3 ± 0.1	237	0.055	2.2 ± 0.1
BF-9	FR rayon/polyester	Needle punched nonwoven	Passive	2.2 ± 0.1	240	0.109	1.5 ± 0.1
BF-10	FR polyester/FR rayon	Stitchbond	Active	0.7 ± 0.1	165	0.236	1.1 ± 0.1
BF-13	Glass fiber core/FR acrylic fiber (core spun yarn)	Knitted	Active	1.4 ± 0.1	165	0.118	1.9 ± 0.1
BF-15	Glass fiber core/FR acrylic fiber	Woven	Active	0.5 ± 0.1	170	0.340	2.1 ± 0.1
BF-16	FR rayon/glass fiber/Poly Lactic Acid (PLA) fiber	Nonwoven	Active	2.9 ± 0.1	290	0.097	1.9 ± 0.2
BF-17	Glass filaments	Woven	Passive	0.2 ± 0.1	150	0.750	0
BF-20	Para-aramid/melamine	Woven	Passive	0.77 ± 0.02	264	0.343	0.2 ± 0.1
BF-21	Para-aramid	Nonwoven	Passive	0.67 ± 0.02	69	0.103	2.1 ± 0.1
BF-22	Meta-aramid/Para-aramid	Woven/non-woven composite	Passive	1.61 ± 0.11	267	0.166	0.9 ± 0.1
BF-23	Cotton/glass fiber	Knit/backcoated	Active/Passive	1.5 ± 0.1	284	0.189	0
BF-24	Polyester	Nonwoven batting	Passive	8.13 ± 1.1	165	0.020	7.6 ± 0.2

^§^ For textile materials, area density is generally expressed as mass per unit area. The standard uncertainty (Type B) [21,22] in measuring area density is about ±5 g/m^2^. A Type B evaluation of standard uncertainty is based on scientific judgment using experience with, and general knowledge of, the behavior and property of relevant materials and instruments.

**Table 3:** Response of FPUF in BF-open flame test configurations.

ID	Structure	12.7 mm (1/2″) thick foam		25.4 mm (1″) thick foam		
		Pass/Fail	Comments	Ignition of FPUF	Time to ignition (s)	Comments
BF-1	Highloft	Fail	Foam ignites due to hole/crack formation in the BF	Yes	40	Foam ignites due to hole/crack formation in the BF
BF-2	Highloft	Pass	Dome formation	Yes	120	Foam ignites due to hole/crack formation in the BF
BF-3	Highloft	Pass	Dome formation	No	-	Dome formation
BF-4	Needle punched nonwoven	Pass	Dome formation	No	-	Dome formation
BF-5	Needle punched nonwoven	Pass	Dome formation	No	-	Dome formation
BF-8	Needle punched flat	Pass	Dome formation	No	-	Dome structure collapses due to formation of hole in the FPUF
BF-9	Needle punched flat	Pass	Dome formation	No	-	Dome structure collapses due to formation of hole in the FPUF
BF-10	Stitchbond	Fail	Foam ignites due to hole/crack formation in the BF	Yes	130	Foam ignites due to hole/crack formation in the BF
BF-13	Knitted	Pass	Dome structure collapses due to formation of hole in the FPUF	Yes	186	Foam ignites due to hole/crack formation in the BF
BF-15	Woven	Pass	Dome structure collapses due to formation of hole in the FPUF	Yes	165	Foam ignites due to hole/crack formation in the BF
BF-16	Nonwoven	Pass	Dome structure collapses due to formation of hole in the FPUF	No	-	Dome structure collapses due to formation of hole in the FPUF
BF-17	Woven glass	Pass	No dome formation, FPUF forms liquid	No	-	No dome formation, FPUF forms liquid
BF-20	Woven	Pass	Dome structure collapses due to formation of hole in the FPUF	No		Dome structure collapses due to formation of hole in the FPUF
BF-21	Nonwoven	Pass	Dome structure collapses due to formation of hole in the FPUF	Yes	240	Dome structure collapses due to formation of hole in the FPUF
BF-22	Woven/nonwoven composite fabric	Pass	Dome structure collapses due to formation of hole in the FPUF	No	-	Dome structure collapses due to formation of hole in the FPUF
BF-23	Knit/backcoated	Pass	No dome formation, FPUF forms liquid	No	-	No dome formation, FPUF forms liquid
BF-24	Polyester batting	Fail	Foam ignites due to hole/crack formation in the BF	Yes	4	Foam ignites due to hole/crack formation in the BF

**Table 4:** Heat transfer data for BFs exposed to BF-open flame test.

ID	Maximum heat flux at unexposed side of barrier at 60 s (kW/m^2^)	Total amount of heat transferred at 60 s (J/cm^2^)	Heat transfer factor (kJ/g)	Visual observations and comments
BF-1	17 ± 2	95 ± 12	61	Thin, fragile char with hole formations
BF-2	8 ± 1	43 ± 1	21	Thermally thick, insulating char
BF-3	6 ± 0.5	35 ± 3	15	Thermally thick, insulating char
BF-4	7 ± 1	40 ± 2	18	Thermally thick, insulating char
BF-5	5 ± 1	30 ± 1	13	Thermally thick, insulating char
BF-8	13 ± 6	73 ± 4	31	Undamaged char
BF-9	14 ± 2	76 ± 10	32	Undamaged char
BF-10	15 ± 1	88 ± 4	53	Thermally thin, cracked barrier
BF-13	29 ± 1	163 ± 4	99	Thermally thin, permeable barrier
BF-15 *	18	104	61	Thermally thin, permeable barrier
BF-16	10 ± 0.3	58 ± 1	20	Undamaged char
BF-17	17 ± 1	98 ± 2	65	Undamaged char
BF-19	16 ± 1	93 ± 7	29	Undamaged char
BF-20	13 ± 2	73 ±16	28	Undamaged char
BF-21	15 ± 3	87 ± 15	126	Undamaged char
BF-22 *	9	52	19	Undamaged char
BF-23	12 ± 1	69 ± 6	24	Undamaged char
BF-24	33 ± 6	190 ± 33	115	Barrier melts and exposes slug calorimeter to the flame

* Single measurements were taken due to the unavailability of test specimens.
